# Metabolic changes in bile acids with pregnancy progression and their correlation with perinatal complications in intrahepatic cholestasis of pregnant patients

**DOI:** 10.1038/s41598-022-22974-8

**Published:** 2023-01-28

**Authors:** Zhixin Ma, Yifeng Liu, Lin Chai, Guochen Jin, Yanni Sun, Shaomin Zhou, Peiyuan Yin, Siwen Wang, Yuning Zhu, Dan Zhang, Shiming Lu, Bo Zhu

**Affiliations:** 1 Key Laboratory of Reproductive Genetics, and Women’s Hospital, School of Medicine, Zhejiang University, Hangzhou, China; 2 Clinical Prenatal Diagnosis Center, Women’s Hospital, School of Medicine, Zhejiang University, Hangzhou, China; 3 Clinical Laboratory of Integrative Medicine, The First Affiliated Hospital of Dalian Medical University, Dalian, China

**Keywords:** Diseases, Medical research, Risk factors

## Abstract

Intrahepatic cholestasis of pregnancy (ICP) is a rare liver disease occurring during pregnancy that is characterized by disordered bile acid (BA) metabolism. It is related to adverse clinical outcomes in both the mother and fetus. Our aim was to evaluate the BA metabolism profiles in different types of ICP and investigate the association between specific BAs and perinatal complications in ICP patients. We consecutively evaluated 95 patients with ICP, in which 53 patients were diagnosed with early-onset ICP (EICP) and 42 patients were diagnosed with late-onset ICP (LICP). Concentrations of 15 BA components were detected using high-performance liquid chromatography tandem mass spectrometry. Clinical information was abstracted from the medical records. The percentage of conjugated bile acids increased in ICP patients. Specifically, taurocholic acid (TCA) accumulated in LICP patients, and glycocholic acid (GCA) predominated in EICP patients. A higher preterm birth incidence was observed among ICP patients. Albumin, total bile acids, total bilirubin and GCA percentage values at ICP diagnosis predicts 83.5% of preterm birth in EICP, and the percentage of TCA in total bile acids at ICP diagnosis predicts 93.2% of preterm birth in LICP. This analysis showed that the BA metabolism profiles of EICP and LICP were distinct. Increased hepatic load was positively correlated with preterm birth in EICP. An elevated TCA percentage in total bile acids provides a biomarker to predict preterm birth in LICP.

## Introduction

Intrahepatic cholestasis of pregnancy (ICP) is a rare liver disease occurring during pregnancy; ICP manifests as pruritus and elevated total bile acid (TBA) concentrations, and affects approximately 0.3–27% of pregnant women worldwide^1,2^. The etiology of ICP is still unknown. Genetics, environmental factors and changes in hormone profiles during pregnancy may all lead to ICP^3^. ICP was once considered a benign situation as the symptoms rapidly resolved after delivery^4^. However, researchers have confirmed that pregnant women with ICP are more susceptible to preeclampsia and gestational diabetes mellitus^5,6^. ICP, especially early-onset ICP (EICP), is associated with adverse perinatal complications, including preterm birth (PTB), stillbirth and even long-term metabolic disorders, which may be attributable to the accumulation of maternal bile acids (BAs) in the fetus and the long exposure time in utero^7–9^.

Maternal serum TBA ≥ 10 μmol/L has diagnostic significance in ICP, and the incidence of poor pregnancy outcomes increases with increasing TBA^4,10,11^. Studies have suggested maternal TBA ≥ 40 μmol/L as a threshold for predicting the occurrence of fetal complications, with each additional 1-μmol/L of TBA increasing the incidence of fetal complications by 1–2%. Furthermore, TBA ≥ 100 μmol/L increased the risk of stillbirth^9,12^. BA metabolism profile analysis has revealed that cholic acid (CA), chenodeoxycholic acid (CDCA) and their glycine and taurine conjugated forms are dominant in TBA of ICP^13,14^. However, studies uncovering the association between specific BAs and the incidence of perinatal complications, particularly preterm birth, which is the most frequent, are still lacking. Since BA metabolism changes with the progression of pregnancy^15^, it is necessary to understand the differences in BA profiles in patients with EICP and late-onset ICP (LICP), as this is critical to understanding why different types of ICP lead to different PTB incidences.

In this study, we first evaluated the characteristics of BA metabolism profiles in sera from patients with different types of ICP using high-performance liquid chromatography tandem mass spectrometry (LC‒MS). Second, according to the unique BA metabolism profiles in ICP patients with PTB, we investigated whether changes in specific BAs were associated with the incidence of PTB in ICP patients, which may help to provide potential valuable biomarkers for early intervention for patients with ICP in clinical practice.

## Materials and methods

### Patients and sample collection

**Figure 1:**
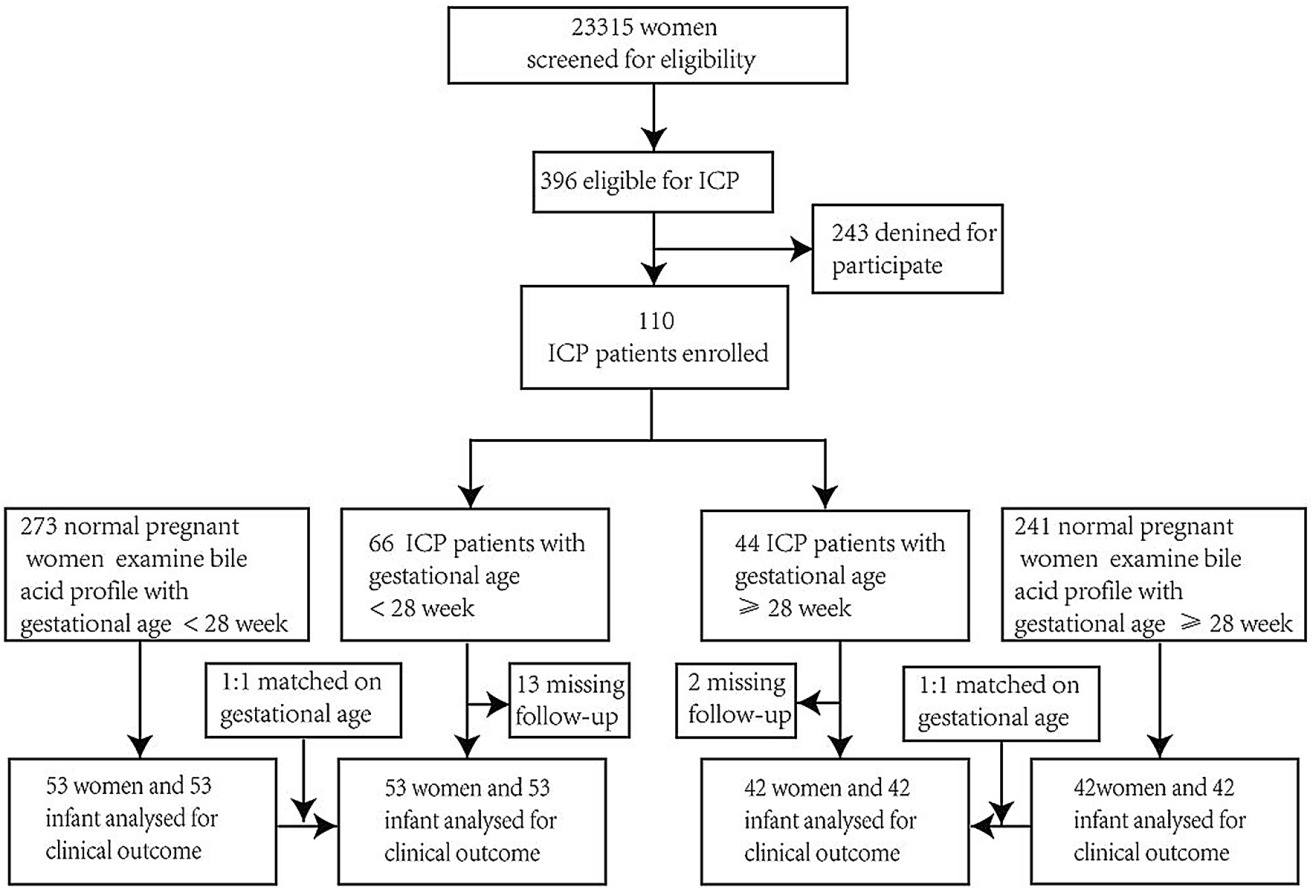
Flowchart showing case enrollment and the completeness of reporting.

Pregnant women admitted to Women’s Hospital, Zhejiang University School of Medicine (ZJUWH) were screened and consulted to participate in the study. Women with ICP were diagnosed according to the ICP guidelines from the Royal College of Obstetricians and Gynecologists edition II^16^. The exclusion criteria were as follows: women who underwent in vitro fertilization and embryo transfer; elevated liver enzymes and low platelet syndrome; multiple pregnancy; preeclampsia; or other hepatic diseases and diseases affecting liver function tests. From July 2019 to May 2020, 53 patients with ICP with gestational age < 28 weeks (defined as the EICP group) and 42 ICP patients with gestational age ≥ 28 weeks (defined as the LICP group) were enrolled in this study. Healthy pregnant women were 1:1 matched for gestational age (defined as the ENC and LNC groups) (Fig. 1). Ursodeoxycholic acid (UDCA) treatment commenced after diagnosis in all patients with ICP. The clinical information of the participants is shown in Table 1. Samples were obtained in a fasting state at primary diagnosis of ICP (pre-UDCA treatment). After centrifugation, the sera were stored at − 80 °C until analysis.

**Table 1:** Baseline characteristics of 190 participants according to ICP diagnosis, 2019–2020.

Variables	NC (n = 95)	ICP (n = 95)	*P* value
Age, median (IRQ), years	29, (27; 32)	30, (28; 34)	ns
TP, median (IRQ), (g/L)	64.5, (62.4; 66.7)	62.9, (60.1; 64.5)	< 0.001
ALB, median (IRQ), (g/L)	35.7, (34.6; 37.4)	34.9, (33.1; 36.6)	0.003
ALT, median (IRQ), U/L	12, (10; 17)	17, (11; 32)	< 0.001
TB, median (IRQ), μmol/L	6.0, (4.8; 7.9)	7, (5.2; 9.3)	0.006
DB, median (IRQ), μmol/L	2.4, (1.9; 3.1)	2.7, (2.1; 4.1)	0.001
IB, median (IRQ), μmol/L	3.5, (2.8; 4.4)	4.1, (2.9; 5.5)	0.033
TBA, median (IRQ), μmol/L	2, (1; 3)	13, (11; 19)	< 0.001
CR, median (IRQ), μmol/L	44.2, (39.6; 48.03)	50.4, (44.1; 56.1)	< 0.001
UREA, median (IRQ), mmol/L	2.45, (2.08; 2.87)	2.70, (2.19;3.16)	0.017
URIC, median (IRQ), μmol/L	235, (210.8; 269.5)	232, (200; 309)	ns
TG, median (IRQ), mmol/L	2.50, (1.99; 3.19)	2.35, (1.84; 3.28)	ns
TCH, median (IRQ), mmol/L	6.27, (5.58; 6.90)	6.08, (5.37; 6.63)	ns
HDL, median (IRQ), mmol/L	1.73, (1.57; 1.93)	1.79, (1.51; 2.02)	ns
GLU, median (IRQ), mmol/L	4.37, (4.19;4.52)	4.34, (4.15;4.57)	ns

### Bile acid measurement

The measurement of BAs was described previously but with minor modifications^15^. Reference standards of cholic acid (CA), chenodeoxycholic acid (CDCA), ursodeoxycholic acid (UDCA), deoxycholic acid (DCA), lithocholic acid (LCA), glycocholic acid (GCA), taurocholic acid (TCA), glycochenodeoxycholic acid (GCDCA), taurochenodeoxycholic acid (TCDCA), glycoursodeoxycholic acid (GUDCA), tauroursodeoxycholic acid (TUDCA), glycodeoxycholic acid (GDCA), taurodeoxycholic acid (TDCA), taurolithocholic acid (TLCA), and glylithocholic acid (GLCA) were commercially available from TRC Inc. (Toronto, Canada) and Sigma–Aldrich (St. Louis, USA).

A total of 100 μl of serum from each participant was mixed with 300 μl of standard solution, quality control acetonitrile and 10 μl of internal standard (IS). After vortexing for 1 min and centrifuging at 13,000 rpm at 4 °C for 15 min, 100 μl of supernatant was applied for LC‒MS analysis. BA analysis was performed by ultra-high-performance liquid chromatography (Agilent Technologies, Santa Clara, USA) coupled to TRIPLE QUAD 5500 (AB Sciex, Framingham, USA). All chromatographic separations were performed with a Poroshell 120 EC-C18 column (1.7 µm, 3 mm × 50 mm) (Agilent Technologies, Santa Clara, USA). The collision energy for each BA is listed in Supplemental Table S1.

### Statistical analysis

PeakView 2.0 (AB Sciex) was used to identify compound identities. Analyst software v1.6.0 (SCIEX, Framingham, USA) was used to quantify confirmed compounds. Student’s t-test or the Mann‒Whitney U test were used to evaluate the difference among groups for independent samples. One-way analysis of variance (ANOVA) or the Kruskal‒Wallis test was used in the case of more than two groups of continuous variables. BA data of participants were averaged according to the gestational ages and then normalized for the generation of a heatmap. Logistic regression was performed with IBM SPSS 18.0. A receiver operating characteristic curve was generated with MedCalc^17^. Statistical analysis was performed using GraphPad Prism 7.0. *P* < 0.05 was considered statistically significant.

### Ethics approval and consent to participate

The study was approved by the Ethics committee of ZJUWH (No. 20170059), in accordance with the Declaration of Helsinki, written informed consent forms were signed by all the participants.

## Results

### Changes in the bile acid metabolism profile in patients with ICP compared with normal pregnant women

**Figure 2:**
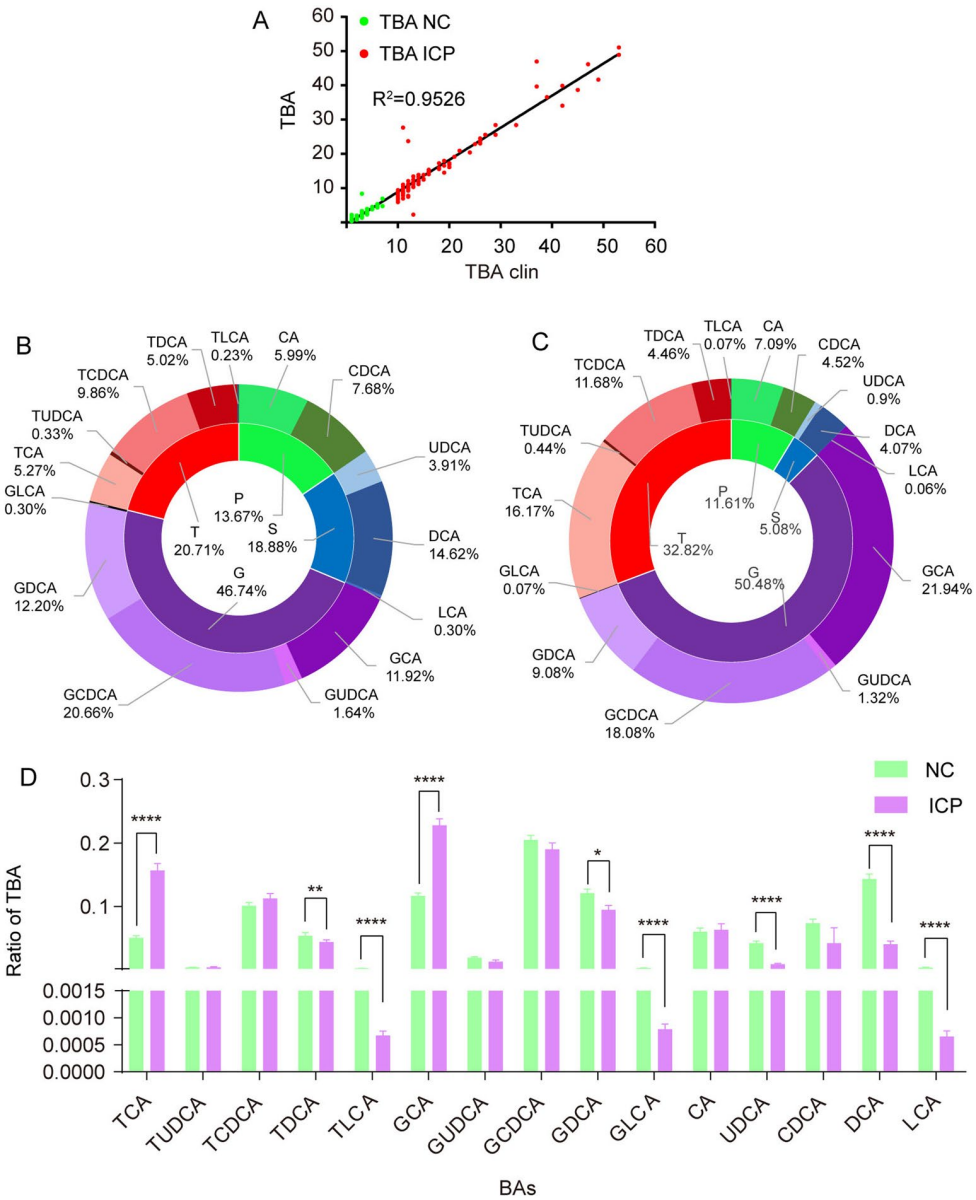
Correlation of TBA detected by cycling enzymatic assay and LC‒MS in all the participants. The green and red dots represent normal pregnant women and women with ICP, respectively (**A**). Pie charts show the percentage variation of BA individuals in TBA between NC (**B**) and ICP patients (**C**), (P: primary BAs, S: secondary BAs, G: glycine conjugated BAs, T: taurine conjugated BAs). T test of BA metabolism changes compared with the normal control (**D**). *P < 0.05; **P < 0.01; ****P < 0.0001.

The TBA detected by LC‒MS was consistent with the results measured by enzymatic cycling assay in all participants (Fig. 2A). In the normal BA pool, primary BAs, secondary BAs, and glycine- and taurine-conjugated BAs represented 13.67%, 18.88%, 46.74% and 20.71%, respectively (Figs. 2B, S1A). In patients with ICP, those four subgroups of BAs represented 11.61%, 5.08%, 50.48% and 32.82% of BAs, respectively (Figs. 2C, S1B). The concentrations of almost all BA components, except UDCA and LCA, surged remarkably (Fig. S1C). Nevertheless, compared with those of healthy pregnant women, the percentages derived from the TBA assay revealed that CA and CDCA remained stable and that DCA, LCA and UDCA all decreased significantly in patients with ICP. Such changes resulted in a significant decrease in the percentages of TLCA, GLCA, TDCA and GDCA and among the conjugated secondary BAs. Since the metabolic pathway of CA was switched to form conjugated CA, the proportions of both GCA and TCA were significantly elevated in ICP patients (Fig. 2D).

### Clinical information and bile acid metabolism characteristics changed with pregnancy progression in normal pregnant women and ICP patients

The clinical information revealed that hepatic and renal function and fatty acid metabolism varied with the increased pregnancy load, regardless of ICP diagnosis. During the second and third trimesters, although indicators remained within the reference range, the liver and renal load increased more in patients with ICP. Total protein (TP), alanine aminotransferase (ALT), total bilirubin (TB), direct bilirubin (DB), indirect bilirubin (IDB), creatinine (CR) and urea (UREA) were elevated in the ICP group. Among all pregnant women, albumin (ALB) showed a decreasing trend as gestational age increased, while CR, uric acid (URIC), triglycerides (TGs), and total cholesterol (TCH) showed an increasing trend with pregnancy progression (Table 2).

**Table 2:** Characteristics of enrolled normal pregnant women and patients with ICP grouped by gestational age.

Variables	ENC (n = 53)	EICP (n = 53)	*P* value	LNC (n = 42)	LICP (n = 42)	*P* value	ENC versus LNC	EICP versus LICP
Age, median (range), years	30, (27; 33)	30, (27; 33)	ns	29, (27; 32)	30, (28; 34)	ns	ns	ns
TP, (g/L)	64, (63; 67)	63, (60; 65)	0.004	64.4, (61.8; 66.73)	62.4, (59.78; 64.58)	0.024	ns	ns
ALB, (g/L)	37, (35; 38)	36, (35; 37)	ns	35.2, (33.65; 36.75)	33.85, (32.2; 35.15)	0.001	0.001	< 0.001
ALT, median (range), U/L	12, (10; 17)	18, (11; 29)	0.012	12.5, (10; 17)	14.5, (11; 32.5)	0.003	ns	ns
TB, median (range), μmol/L	5.6, (4.45; 7.55)	7.5, (5.55; 9.1)	0.032	6.2, (4.98; 8.05)	6.2, (5.08; 9.85)	ns	ns	ns
DB, median (range), μmol/L	2.4, (1.7; 2.95)	2.8, (2.05; 3.85)	0.026	2.4, (2.1; 3.23)	2.6, (2.18; 4.23)	0.017	ns	ns
IB, median (range), μmol/L	3.4, (2.8; 4.25)	4.5, (3; 5.75)	0.048	3.75, (2.8; 4.55)	3.55, (2.78; 5.1)	ns	ns	ns
TBA, median (range), μmol/L	2, (1; 3)	13, (11; 21.5)	< 0.001	2, (2; 3)	13, (10.75; 15.25)	< 0.001	ns	0.029
CR, median (range), μmol/L	43, (38.25; 47.25)	48.15, (42.48; 51.68)	0.001	45.6, (42.3; 49.85)	53.9, (48.4; 64.95)	< 0.001	0.010	< 0.001
UREA, median (range), mmol/L	2.49, (2.14; 2.81)	2.65, (2.14; 2.93)	ns	2.44, (1.97; 2.93)	2.87, (2.33; 3.45)	0.011	ns	0.035
URIC, median (range), μmol/L	225, (205.5; 253.5)	215, (187; 238)	ns	255, (222; 300)	288, (233; 345.5)	ns	0.001	< 0.001
TG, median (range), mmol/L	2.15, (1.67; 2.76)	2.08, (1.65; 2.60)	ns	3.09, (2.44; 3.72)	3.07, (2.36; 4.22)	ns	< 0.001	< 0.001
TCH, median (range), mmol/L	5.94, (5.46; 6.63)	5.9, (5.05; 6.38)	ns	6.7, (5.79; 7.22)	6.29, (5.5; 7.3)	ns	0.011	0.016
HDL, median (range), mmol/L	1.73, (1.58; 1.93)	1.86, (1.6; 2.05)	ns	1.7, (1.55; 1.94)	1.57, (1.39; 1.98)	ns	ns	0.029
GLU, median (range), mmol/L	4.39, (4.19; 4.52)	4.29, (4.06; 4.55)	ns	4.37, (4.20; 4.53)	4.35, (4.18; 4.65)	ns	ns	ns

**Figure 3:**
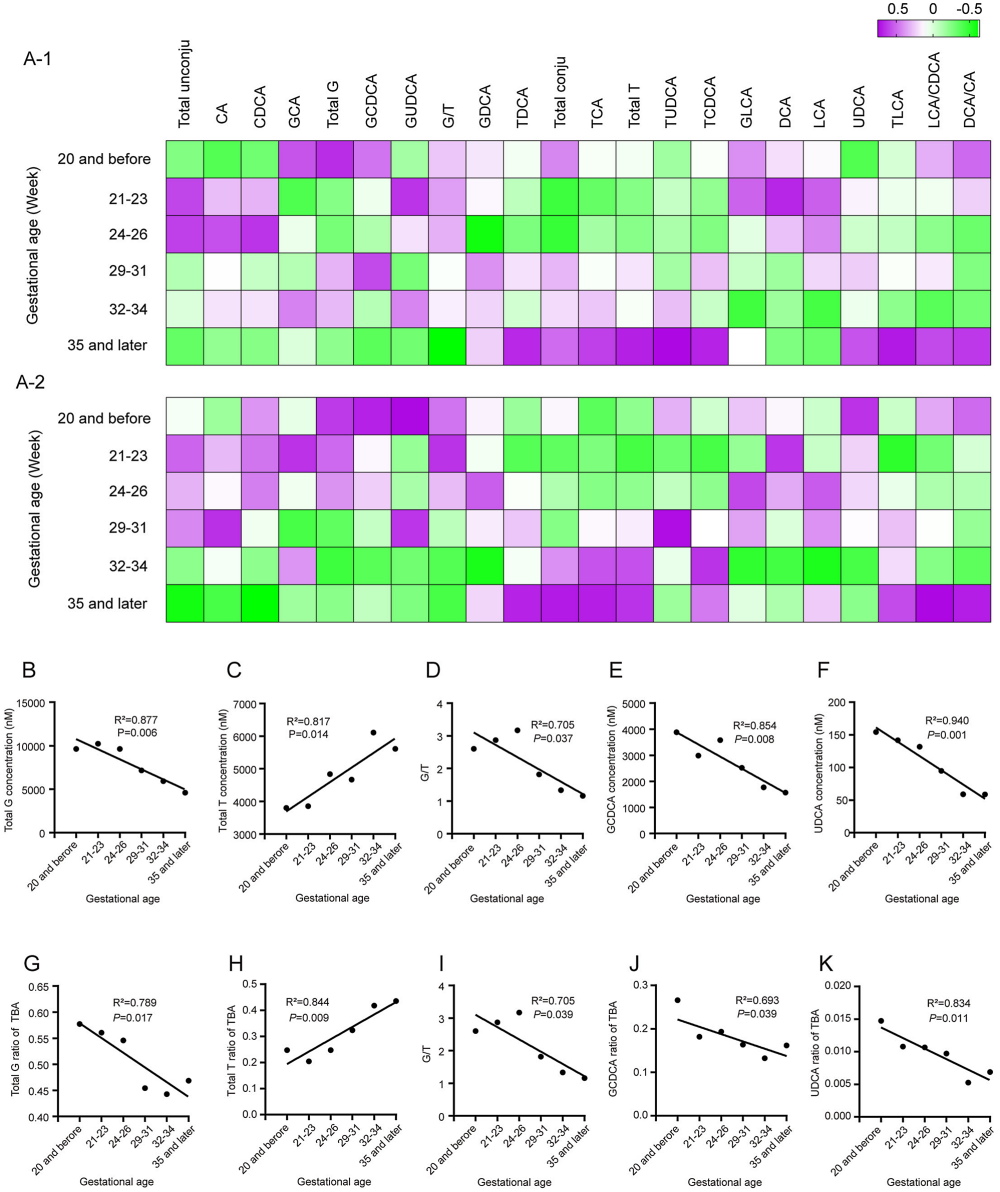
Heatmap showing the BA metabolism profiles that changed with pregnancy advance in normal (**A-1**) and ICP (**A-2**). Total unconju: total unconjugated BAs; Total conju: total conjugated BAs; Total G: total glycine conjugated BAs; Total T: total taurine conjugated BAs; G/T: Total G/Total T. Linear changes in concentrations and percentages of total G (**B**, **G**), total T (**C**, **H**), G/T (**D**, **I**), GCDCA (**E**, **J**), and UDCA (**F**, **K**) at different diagnostic time points in ICP patients.

To further understand the BA metabolism profiles of different types of ICP, we examined the BA spectrum in normal pregnant women. Although TBA homeostasis was sustained throughout the second and third trimesters, changes in BA components occurred. Specifically, in the second trimester, unconjugated BAs predominated in serum BA pools, whereas conjugated components progressively increased as the pregnancy advanced in the third trimester (Figs. 3A-1, S2A). Interestingly, a similar trend was also observed in patients with ICP. The changes in certain BAs were significantly correlated with gestational age (Figs. 3A-2, S2B). Both the concentrations and ratios of total G and GCDCA in TBA, UDCA and G/T decreased, whereas total T increased with increasing gestational age (Fig. 3B–K). It is worth noting that such changes indicate that there is a discrepancy between EICP and LICP in BA metabolism profiles.

### Characteristics of bile acid metabolism profiles in early-onset ICP (EICP) and late-onset ICP (LICP)

**Figure 4:**
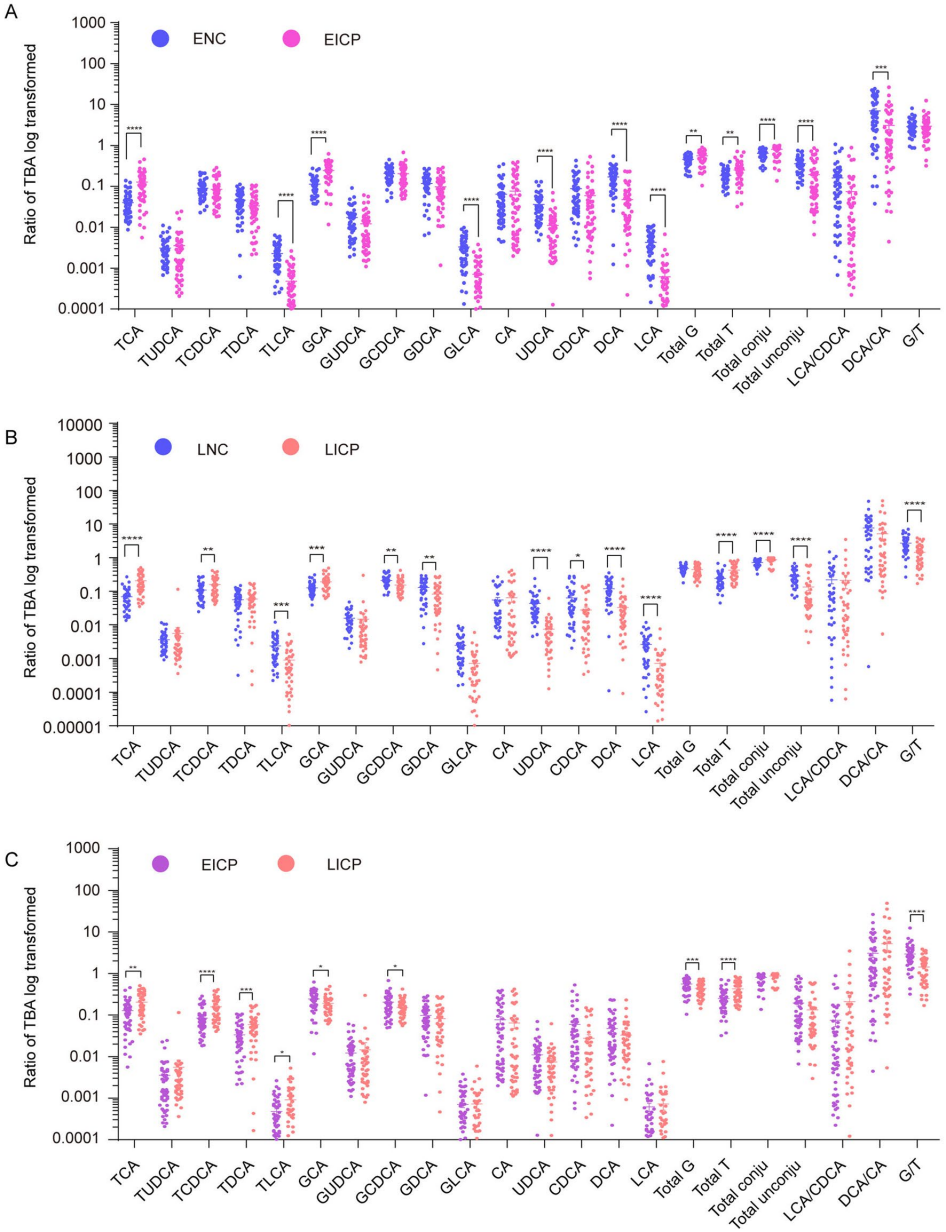
Changes in BA metabolism profiles in EICP (**A**) and LICP (**B**) compared with normal pregnant women. Different BA metabolism profiles between EICP and LICP (**C**), BAs are represented as the percentage of TBA and log transformed. *P < 0.05; **P < 0.01; ***P < 0.001; ****P < 0.0001.

Virtually, the BA profiles in EICP and LICP were not only different from those of normal pregnant women but also distinguishable from each other. In EICP serum, the concentrations of 14 BAs were increased, with LCA being the exception. LCA/CDCA and DCA/CA were significantly decreased (Fig. S3A). In LICP serum, the concentrations of 13 BAs were elevated, with LCA and UDCA being the exceptions, and that of G/T was decreased (Fig. S3B). The percentages of TCA, GCA, total T and conjugated BAs were elevated. The percentages of TLCA, GLCA, UDCA, DCA, LCA and unconjugated BAs were decreased in both the EICP and LICP groups (Fig. 4A,B). Compared with patients with EICP, those with LICP had lower concentrations of unconjugated BAs, including UDCA, CDCA and DCA. Conjugated BA levels, including TCDCA, GCDCA, GCA and total G, were decreased in patients with LICP (Fig. S3C). However, the percentages of GCA, GCDCA and total G were decreased, while the percentages of TCA, TCDCA, TDCA, TLCA and total T were increased, which resulted in decreased G/T in LICP (Fig. 4C).

### Incidence of perinatal complications among patients with ICP compared with normal pregnant women

In Table 3, we show that patients with ICP had a higher incidence of preeclampsia and fetal growth restriction, but the difference was not significant. In addition, the EICP group tended to further develop gestational diabetes mellitus, although the difference was not significant. Of note, exposure to ICP was associated with a higher PTB rate (EICP: 24.53% vs. ENC: 3.77%; LICP: 16.67% vs. LNC: 0%), fewer gestational weeks (EICP: 38, (36, 38) vs. ENC: 39, (38, 40); LICP: 38, (37, 39) vs. LNC: 39, (38, 40), especially in the EICP group (EICP: 38, (36, 38) vs. LICP: 38, (37, 39); however, no difference in fetal birthweight was observed among participants.

**Table 3:** Clinical outcomes of early and late onset ICP compared with normal pregnancy.

Sample	ENC (n = 53)	EICP (n = 53)	*P* value	LNC (n = 42)	LICP (n = 42)	*P* value	EICP versus LICP
Gestational week, median (IQR)	39, (38; 40)	38, (36; 38)	< 0.001	39, (38; 40)	38, (37; 39)	0.008	0.016
Furtherly accompanied with GDM	1, (1.89%)	3, (5.66%)	ns	NA	NA	NA	NA
Pre-eclampsia	0, (0%)	2, (3.77%)	ns	0, (0%)	1, (2.38%)	ns	ns
Fetal growth restriction	0, (0%)	1, (1.89%)	ns	1, (2.38%)	4, (9.52%)	ns	ns
Birth weight, median (IQR), g	3330, (3080; 3650)	3010, (2673; 3348)	ns	3390, (3025; 3465)	3215, (2750; 3618)	ns	ns
Preterm birth, n (%)	2, (3.77%)	13, (24.53%)	0.002	0 (0%)	7, (16.67%)	0.005	ns

### Potential increase in predictive values of bile acids for preterm birth

**Figure 5:**
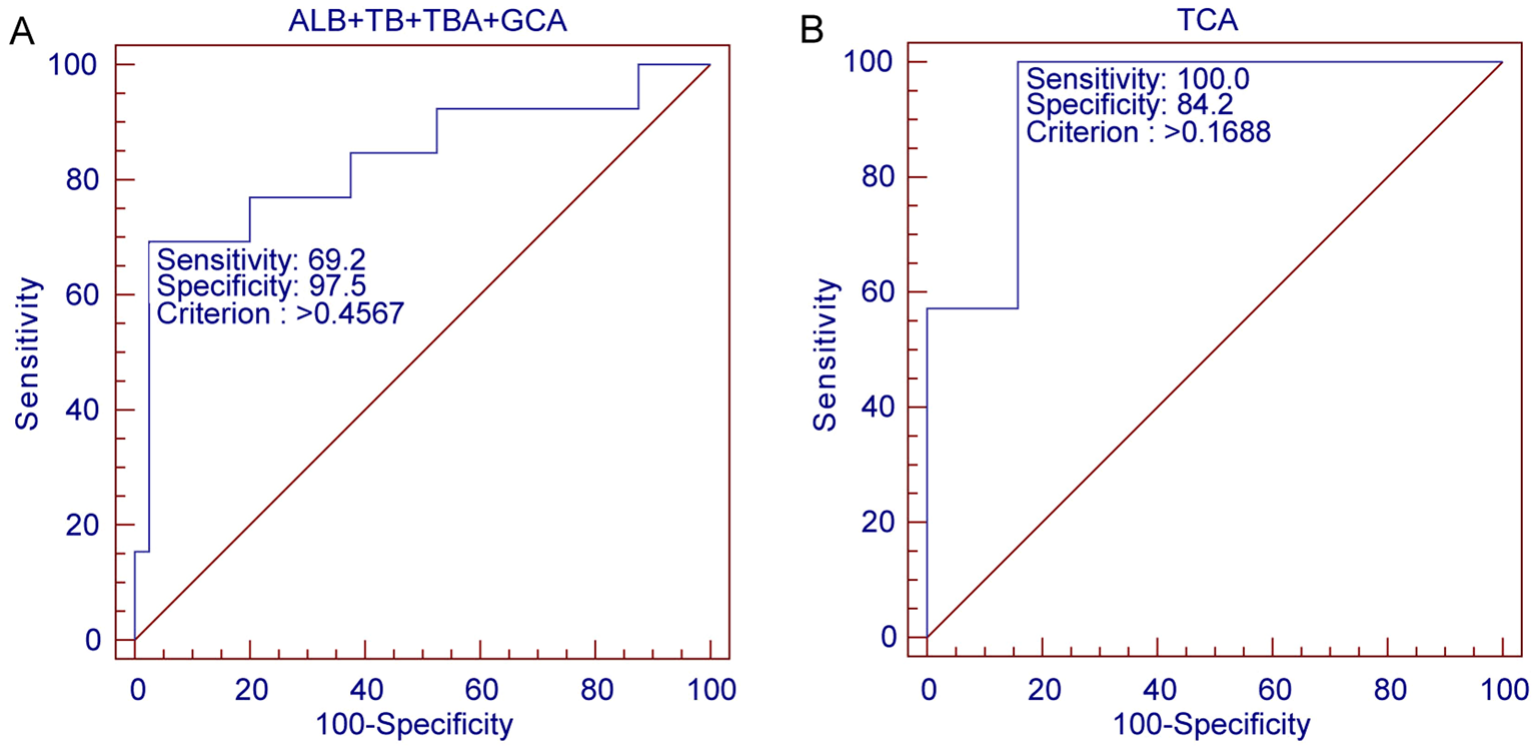
Receiver operating characteristic curves of TBA + GCA percentage of TBA + ALB + TB for the prediction of PTB in EICP (**A**). Receiver operating characteristic curves of TCA percentage of TBA for the prediction of PTB in LICP (**B**).

Logistic regression revealed that elevated TBA, a high percentage of GCA in TBA, and increased serum ALB, TB, DB and IB levels were risk factors associated with PTB in the EICP group (Table S2). The single-parameter models to predict PTB had similar areas under the curve (Table S3). The inclusion of TBA, GCA percentage in TBA, ALB and TB into one model significantly increased the AUC to 0.84 (95% CI 0.71 to 0.92, *P* < 0.001) (Fig. 5A). In the LICP group, the single TCA percentage in TBA obtained an AUC of 0.93 (95% CI 0.76 to 0.99, *P* < 0.001) (Fig. 5B) (Table S4). Although renal function-related parameters HDL, TCDCA and total T were also related to PTB in LICP (Table S5), combining them with TCA did not improve the AUC and 95% CI in multiple prediction models.

## Discussion

ICP has been reported to increase perinatal complications, including PTB and even intrauterine fetal death. This study was designed to study the metabolic features of BAs in patients with ICP during the second and third trimesters and then aimed to discover potential biochemical markers to screen for PTB. Our data are in line with previous findings that ICP patients are more likely to experience PTB and that increased serum TBA concentrations are correlated with a higher risk of PTB. More importantly, we further demonstrated that an elevated percentage of GCA in TBA and ALB, TBA and TB is related to an elevated PTB rate among patients with EICP. Increased TCA percentages can be used to predict PTB in the LICP group.

Maternal circular TBA elevation has been proven to be positively correlated with the incidence of fetal complications, including PTB and stillbirth in ICP^18,19^. However, the mechanism of ICP-induced PTB is still uncertain. This effect seems to be related to the accumulation of BAs in the fetal compartment and the increased reaction of the myometrium to BAs. After exposure to BAs from patients with ICP, myometrial cells isolated from women without ICP had increased sensitivity to oxytocin^20^. The oxytocin signaling pathway is abnormally activated, leading to the upregulation of oxytocin receptor expression in ICP^21^. Except for TBAs, certain BA components are also related to PTB. Deng and his colleagues demonstrated that 0.5% CA in the diet led to a PTB rate of 50% in mice, and an increased incidence of PTB meconium-stained amniotic fluid was observed in 100% of lambs that received CA infusion^22,23^. Here, we show that increased GCA is related to PTB in EICP and that high TCA is associated with the incidence of PTB in LICP. The different CA-conjugated BA forms related to different types of ICP may be ascribed to metabolic changes in the BA profile throughout pregnancy.

The BA metabolism profile changes during the second and third trimesters in the healthy state^15^. Such changes are also more obvious in ICP, with high concentrations of glycine-conjugated BAs observed in EICP, and taurine-conjugated BAs increased in LICP. However, substantial changes in the percentages of TBA are blurred by the seemingly raised concentrations. BA metabolism in both EICP and LICP is represented by decreased percentages of unconjugated individuals, suggesting disordered BA metabolic flux in ICP. The occurrence of ICP may be due to the hindered transformation of primary to secondary BAs, which results in the accumulation of conjugated CA and CDCA. In addition, it can be explained in the reverse manner that excessive production of conjugated BAs prevents the primary BAs from converting to secondary BAs. Further research is warranted to elucidate the cause-and-effect relationship.

UDCA has been recommended as the first-line medication in ICP treatment for decades^4^. However, its efficacy is controversial. Studies have shown that patients with ICP experience obvious pruritus relief after UDCA treatment, with lowered biochemical parameters^24^. The impact of UDCA administration on the fetus has not been evaluated with adequately powered research, although a recent randomized controlled trial based on a large population indicated that UDCA treatment benefited patients with ICP in terms of perinatal outcomes^25^. A meta-analysis showed that UDCA was likely to reduce adverse pregnancy outcomes in patients with ICP; furthermore, there was a significant reduction in TBAs in both maternal serum and umbilical cord serum and a qualitative variation in the serum BA pool, with the hydrophobic BA component reduced in the pool^26,27^. However, the recent PITCHES trial revealed that TBA was not reduced in the UDCA group compared with the placebo group in patients with ICP^25^. Indeed, after 500 mg–2 g/day intake of UDCA medication, serum UDCA, GUDCA and TUDCA accounted for almost 60% of serum TBA^28^. In clinical practice, the vast majority of PTB in women with ICP occurs in the late pregnancy period and is often iatrogenic. The reason for iatrogenic PTB in pregnant women with ICP is predominantly elevated TBA. Thus, it might be more reasonable to understand the BA profiles more comprehensively after UDCA treatment with the aid of LC‒MS, not just TBA. Herein, we established a model to provide GCA and TCA as PTB predictive biomarkers. Moreover, changes in GCA and TCA may be helpful for evaluating UDCA treatment efficacy. Fully understanding the BA spectrum may also contribute to the decision making of early induction in late pregnancy to avoid the incidence of iatrogenic PTB.

Furthermore, normal hepatic function is essential for the maintenance of a normal pregnancy^29^. Liver size enlarges gradually as pregnancy progresses to meet the nutritional and metabolic requirements of the growing fetus and pregnant woman, and this is accompanied by increases in biochemical parameters, representing the liver’s compensation during pregnancy^30,31^. However, when the compensation can no longer meet these demands, the risk of PTB is increased. Thus, PTB may be a protective mechanism that ensures the survival of the pregnant woman when her liver is no longer able to compensate. In EICP, elevated ALB, TBA, TB and GCA percentages taken together predict the risk of PTB, suggesting that the increased liver load in the second trimester is positively correlated with PTB. The patient’s hepatic function state at the ICP diagnostic time point has an important role in determining her further perinatal and offspring outcomes following the occurrence of PTB.

## Conclusion

In conclusion, we found that patients with different types of ICP have distinct serum BA profiles. BA metabolism is not disorganized in ICP. In fact, it is related to gestational age. Moreover, we revealed previously unnoticed variations in BAs, which may help to explain the underlying mechanism of ICP development. Increased ALB, TBA, TB and GCA percentage of TBA are useful to predict PTB in EICP, suggesting that a high hepatic load in the second trimester increases PTB risk. In addition, an elevated TCA percentage might be used to predict PTB in patients with LICP.

## Supplementary Information

Supplementary Information.

## Data Availability

All the data are available to interested researchers upon reasonable request. Requests for access to data should be made to the first author with e-mail: 11418122@zju.edu.cn.

## Abbreviations

ICP: Intrahepatic cholestasis of pregnancy
EICP: Early-onset intrahepatic cholestasis of pregnancy
LICP: Late-onset intrahepatic cholestasis of pregnancy
BAs: Bile acids
PTB: Preterm birth
TBA: Total bile acids
TCA: Taurocholic acid
GCA: Glycocholic acid
CA: Cholic acid
CDCA: Chenodeoxycholic acid
LC–MS: Liquid chromatography tandem mass spectrometry
ENC: EICP control group
LNC: LICP control group
UDCA: Ursodeoxycholic acid
CA: Cholic acid
CDCA: Chenodeoxycholic acid
DCA: Deoxycholic acid
LCA: Lithocholic acid
GCDCA: Glycochenodeoxycholic acid
TCDCA: Taurochenodeoxycholic acid
GUDCA: Glycoursodeoxycholic acid
TUDCA: Tauroursodeoxycholic acid
GDCA: Glycodeoxycholic acid
TDCA: Taurodeoxycholic acid
TLCA: Taurolithocholic acid
GLCA: Glylithocholic acid
IS: Internal standard
UHPLC: Ultra-high performance liquid chromatography
ANOVA: One-way analysis of variance
ROC: Receiver operating characteristic curve
TP: Total protein
ALT: Alanine aminotransferase
TB: Total bilirubin
DB: Direct bilirubin
IDB: Indirect bilirubin
CR: Creatinine
UREA: Urea
ALB: Albumin
URIC: Uric acid
TG: Triglyceride
TCH: Total cholesterol
