# Supplementary material for: Metabolic changes in bile acids with pregnancy progression and their correlation with perinatal complications in intrahepatic cholestasis of pregnant patients
Source: Sci Rep. 2023 Jan 28;13:1608. doi: 10.1038/s41598-022-22974-8 (PMC9884190; doi:10.1038/s41598-022-22974-8)
Supplement: Supplementary file 1 — Supplementary Information. [file 41598_2022_22974_MOESM1_ESM.docx]

**Figure legends**

Figure. S1 Bar charts showed the percentage variation of bile acids respectively in TBA between normal pregnant women (A) and patients with ICP (B), (Primary: primary bile acid, Secondary: secondary bile acids, G conjugated: glycine conjugated bile acid, T conjugated: taurine conjugated bile acids). Serum concentrations of 13 bile acids increased significantly in ICP group except UDCA and LCA (C). ****:*P* < 0.0001.

Figure. S2 Heatmap showed the bile acids metabolism profiles changing in each week with the pregnancy progression in normal (A) and ICP groups (B), (Total unconju: total unconjugated bile acids, Total conju: total conjugated bile acids, Total G: total glycine conjugated bile acid, Total T: total taurine conjugated bile acids. G/T: Total G/Total T). In normal pregnant women, 17-19 and 36-38 gestation weeks were grouped together due to the number of the participants. ICP patients with the gestational age of 11-19, 35-36, 38-40 weeks were treated in the same way.

Figure. S3 Changes of bile acids metabolism profiles in early-onset ICP (A) and late-onset ICP (B) compared with normal pregnant women during the similar gestational age. Difference of bile acids metabolism profiles between early and late-onset ICP (C). Bile acids were represented in the concentration (nM) and were log transformed. *: *P* < 0.05; **: *P* < 0.01; ***: *P*<0.001 ****: *P* < 0.0001.

**Figure. S1**


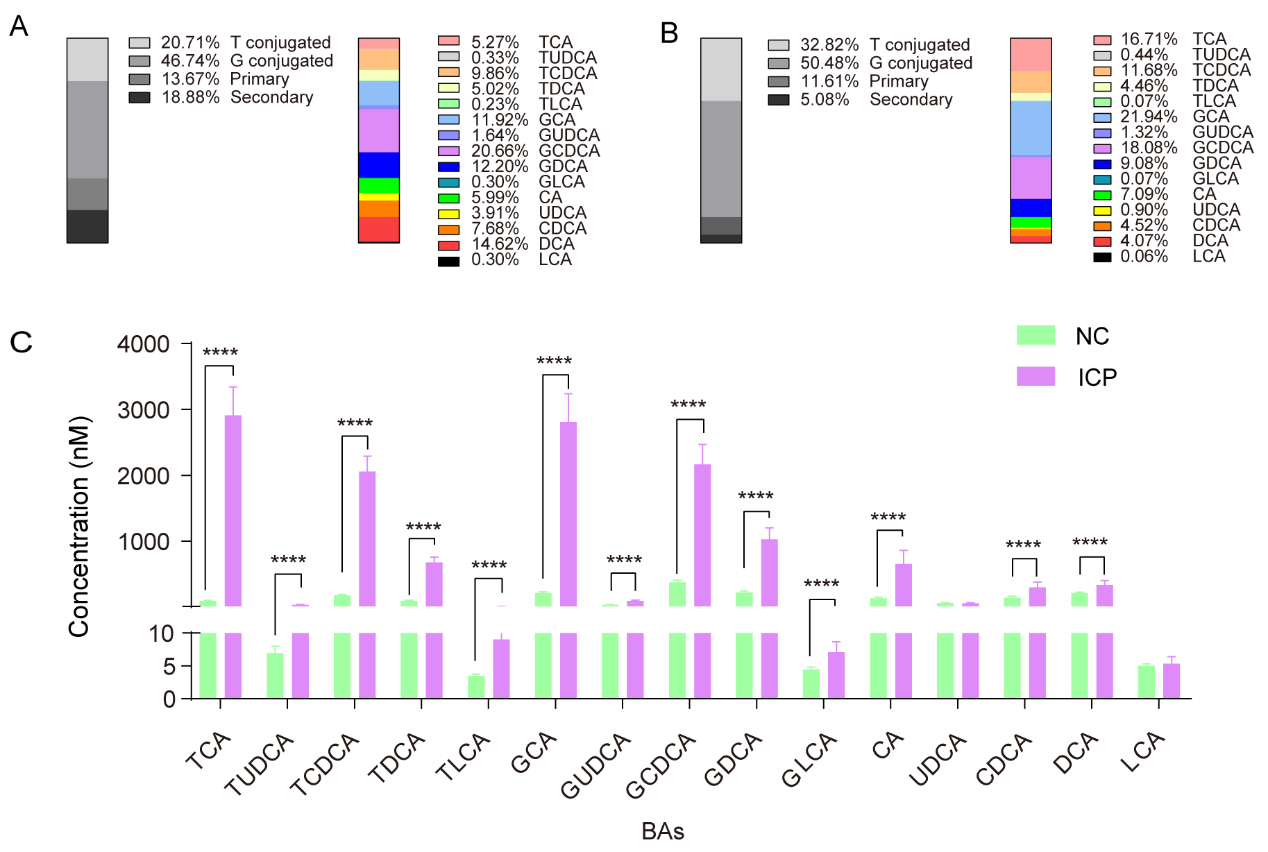


**Figure. S2**


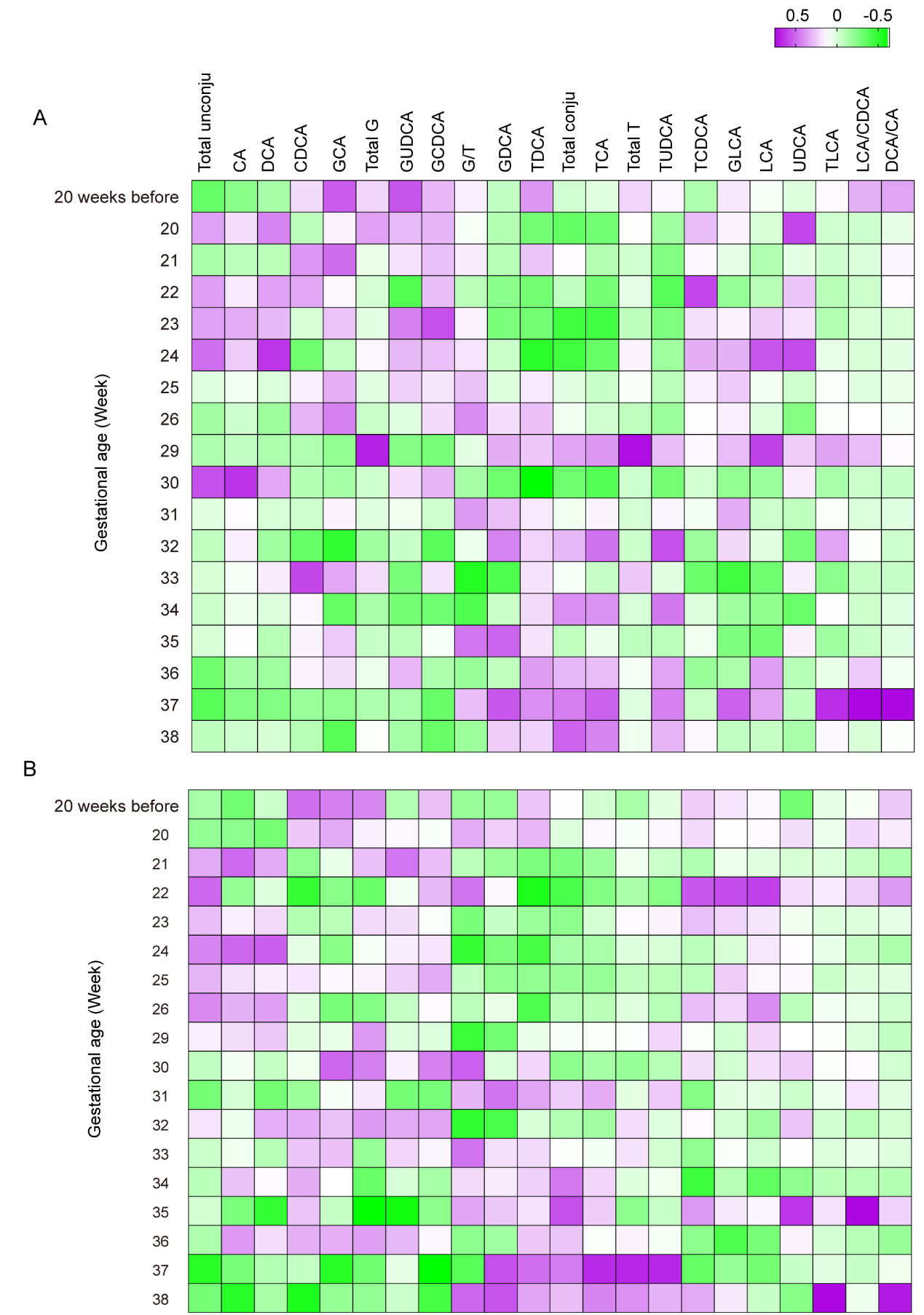


**Figure. S3**


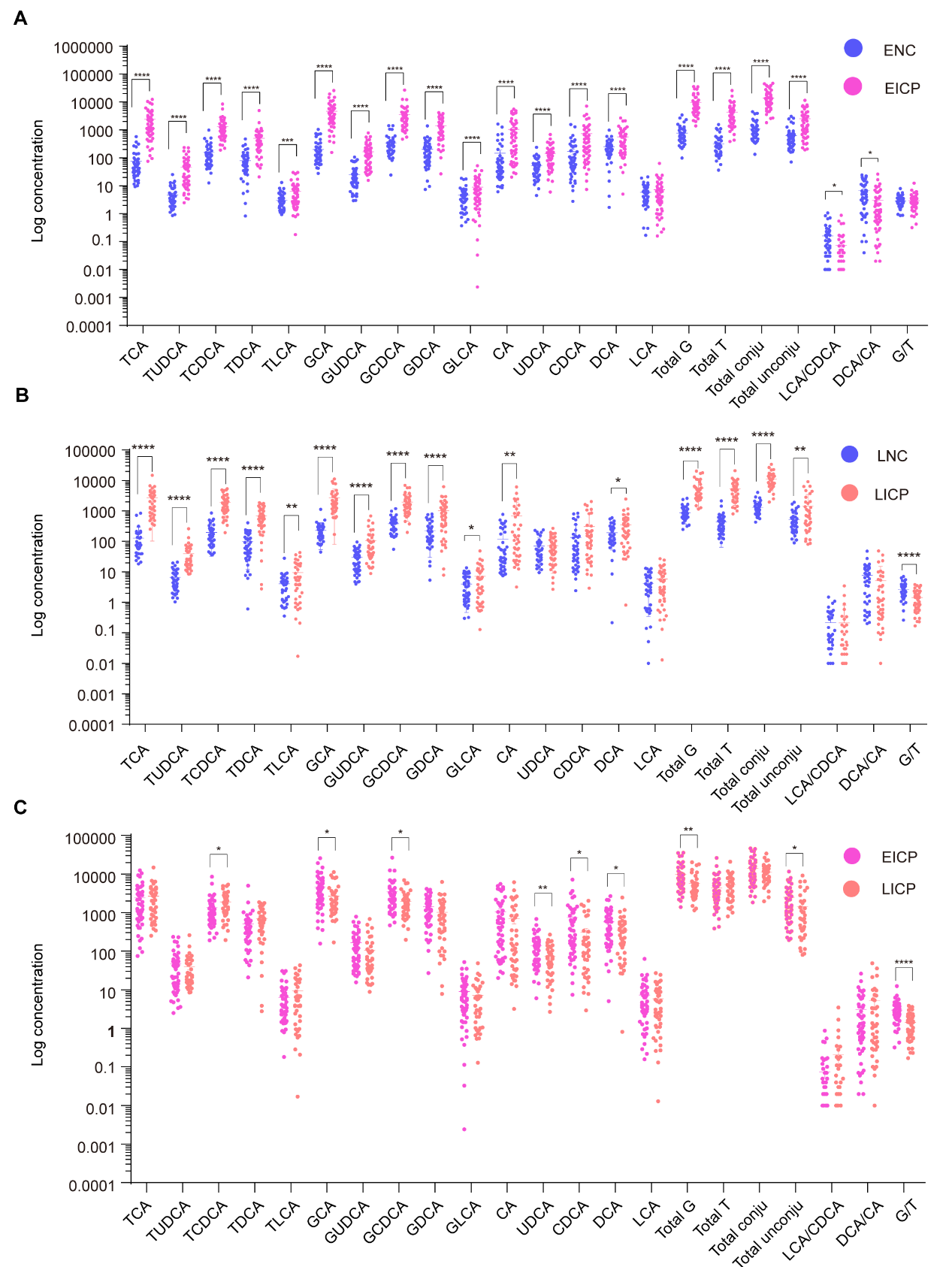


Table S1. The CE, CXP, Q1, Q3 and retention time for each bile acid.

| Bile acid | Retention time(min) | Q1 Mass (m/z) | Q3 Mass(m/z) | Cone(V) | Collision(V) |
| --- | --- | --- | --- | --- | --- |
| TUDCA | 1.17 | 514.3 | 80.0 | 30 | 60 |
| TCA | 1.19 | 514.3 | 80.0 | 30 | 60 |
| GCA | 1.52 | 464.3 | 74.0 | 30 | 35 |
| GUDCA | 1.54 | 448.3 | 74.0 | 30 | 35 |
| TCDCA | 1.67 | 514.3 | 80.0 | 30 | 60 |
| TDCA | 1.82 | 498.3 | 80.0 | 30 | 60 |
| GCDCA | 2.30 | 448.3 | 74.0 | 30 | 35 |
| CA | 2.47 | 407.3 | 407.3 | 30 | 20 |
| GDCA | 2.48 | 448.3 | 74.0 | 30 | 32 |
| TLCA | 2.55 | 482.3 | 80.0 | 30 | 58 |
| UDCA | 2.60 | 391.2 | 391.2 | 30 | 30 |
| CDCA | 3.59 | 391.2 | 391.2 | 30 | 20 |
| GLCA | 3.61 | 432.2 | 74.0 | 30 | 35 |
| DCA | 3.75 | 391.2 | 391.2 | 30 | 20 |
| LCA | 4.62 | 375.3 | 375.3 | 30 | 20 |

Table S2. Clinical data and bile acids characteristics of EICP patients with preterm birth and non-preterm birth.

| Variables | Normal | Preterm birth | *P* value |
| --- | --- | --- | --- |
| Gestational age, median (IQR), weeks | 23, (20, 25) | 24, (21, 25) | 0.356 |
| Age, median (IQR), years | 30, (28, 33) | 28, (26, 36) | 0.885 |
| TP, median (IQR), g/L | 63, (60.2, 64.6) | 63.4, (61.15, 63.85) | 0.362 |
| ALB, median (IQR), g/L | 35.6, (34.5, 36.8) | 36.9, (35.7, 38.2) | 0.008 |
| ALT, median (IQR), U/L | 18, (12, 32) | 13, (7, 30.5) | 0.564 |
| AST, median (IQR), U/L | 18, (16, 27) | 18, (14, 23.75) | 0.878 |
| TB, median (IQR), μmol/L | 7.1, (5.2, 8.8) | 9.1, (6.2, 20.35) | 0.001 |
| DB, median (IQR), μmol/L | 2.7, (1.9, 3.8) | 3.5, (2.3, 8.05) | 0.003 |
| IB, median (IQR), μmol/L | 4.1, (2.9, 5.6) | 5.6, (4.15, 11.65) | 0.002 |
| CR, median (IQR), μmol/L | 48.3, (43, 51.6) | 46.15, (41.13, 51.55) | 0.385 |
| UREA, median (IQR), mmol/L | 2.64, (2.13, 2.89) | 2.88, (2.16, 3.37) | 0.149 |
| URIC, median (IQR), μmol/L | 214, (187, 236) | 234, (188, 289) | 0.151 |
| TG, median (IQR), mmol/L | 2.16, (1.71, 2.73) | 2.04, (1.425, 2.725) | 0.867 |
| TCH, median (IQR), mmol/L | 5.9, (5.07, 6.49) | 5.62, (4.8, 6.155) | 0.675 |
| HDL, median (IQR), mmol/L | 1.9, (1.68, 2.03) | 1.72, (1.395, 2.085) | 0.166 |
| GLU, median (IQR), mmol/L | 4.27, (4.03, 4.53) | 4.37, (4.115, 4.54) | 0.314 |
| TCA, median (IQR) | 0.1077, (0.04682, 0.1574) | 0.1278, (0.09738, 0.2034) | 0.697 |
| TUDCA, median (IQR) | 0.001546, (0.000879, 0.004411) | 0.001066, (0.0003965, 0.004107) | 0.361 |
| TCDCA, median (IQR) | 0.06554, (0.04899, 0.1002) | 0.05369, (0.03124, 0.08869) | 0.187 |
| TDCA, median (IQR) | 0.0241, (0.01399, 0.04831) | 0.02227, (0.004174, 0.03895) | 0.135 |
| TLCA, median (IQR) | 0.000282, (0.000126, 0.000699) | 0.000142, (0.0000773, 0.000372) | 0.154 |
| GCA, median (IQR) | 0.2182, (0.152, 0.2777) | 0.3629, (0.2304, 0.4044) | 0.007 |
| GUDCA, median (IQR) | 0.006573, (0.00384, 0.01737) | 0.005454, (0.002157, 0.0138) | 0.495 |
| GCDCA, median (IQR) | 0.1814, (0.1171, 0.2524) | 0.1683, (0.1046, 0.2978) | 0.465 |
| GDCA, median (IQR) | 0.08588, (0.04658, 0.1437) | 0.06415, (0.03598, 0.1243) | 0.119 |
| GLCA, median (IQR) | 0.000384, (0.000078, 0.00124) | 0.000338, (0.00006475, 0.000447) | 0.159 |
| CA, median (IQR) | 0.03075, (0.005286, 0.1288) | 0.00869, (0.005467,0.08236) | 0.476 |
| UDCA, median (IQR) | 0.009219, (0.003195, 0.01682) | 0.004701, (0.002676, 0.009287) | 0.208 |
| CDCA, median (IQR) | 0.02328, (0.008218, 0.06886) | 0.01186, (0.005676, 0.0329) | 0.980 |
| DCA, median (IQR) | 0.01944, (0.0109, 0.07412) | 0.01505, (0.009984, 0.03529) | 0.088 |
| LCA, median (IQR) | 0.000344, (0.000095, 0.000855) | 0.000156, (0.000136, 0.0004465) | 0.152 |
| Total G, median (IQR) | 0.5652, (0.3997, 0.6988) | 0.6369, (0.6072, 0.7216) | 0.108 |
| Total T, median (IQR) | 0.2166, (0.1251, 0.3044) | 0.249, (0.1588, 0.2934) | 0.527 |
| Total conju, median (IQR) | 0.8926, (0.6195, 0.9467) | 0.927, (0.8569, 0.9682) | 0.375 |
| Total unconju, median (IQR) | 0.1074, (0.05332, 0.3805) | 0.07299, (0.03184, 0.1431) | 0.375 |
| G/T, median (IQR) | 2.566, (1.89, 3.592) | 2.817, (1.94, 4.129) | 0.209 |
| Conju/Unconju, median (IQR) | 8.309, (1.628, 17.75) | 12.7, (6.872, 31.25) | 0.926 |
| CA/CDCA, median (IQR) | 1.079, (0.4678, 2.201) | 1.189, (0.527, 3.217) | 0.865 |
| DCA/CA, median (IQR) | 1.128, (0.2876, 3.902) | 1.018, (0.279, 2.227) | 0.734 |
| LCA/CDCA, median (IQR) | 0.01101, (0.001193, 0.04734) | 0.01486, (0.00365, 0.07296) | 0.329 |
| TBA, umol/L median (IQR) | 13, (11, 21) | 20, (12.5, 39.5) | 0.001 |

Table S3. Clinical data and bile acids characteristics of LICP patients with preterm birth and non-preterm birth.

| Variables | Normal | Preterm birth | *P* value |
| --- | --- | --- | --- |
| Gestational age, median (IQR), weeks | 31, (30, 34) | 31, (29, 34) | 0.374 |
| Age, median (IQR), years | 31, (28, 33) | 38, (28, 38) | 0.073 |
| TP, median (IQR), g/L | 61.4, (59.6, 64.3) | 64.2, (63.2, 65.5) | 0.089 |
| ALB, median (IQR), g/L | 34, (32.7, 34.8) | 34.1, (29.3, 35.1) | 0.181 |
| ALT, median (IQR), U/L | 12, (9, 23) | 15, (10, 36) | 0.410 |
| AST, median (IQR), U/L | 17.5, (13.75, 21) | 20, (14,35) | 0.941 |
| TB, median (IQR), μmol/L | 5.7, (5, 7.9) | 5.8, (5, 13) | 0.166 |
| DB, median (IQR), μmol/L | 2.4, (2.1, 3) | 2.8, (2, 5) | 0.123 |
| IB, median (IQR), μmol/L | 3.4, (2.9, 4.4) | 2.8, (2.6, 7.4) | 0.548 |
| CR, median (IQR), μmol/L | 51.7, (46.25, 58.6) | 78.8, (53.3, 86.7) | 0.005 |
| UREA, median (IQR), mmol/L | 2.59, (1.918, 3.028) | 3.08, (2.69, 5.34) | 0.030 |
| URIC, median (IQR), μmol/L | 248, (211, 343.3) | 394.5, (236, 430.5) | 0.021 |
| TG, median (IQR), mmol/L | 2.9, (2.413, 3.785) | 3.78, (2.82, 4.645) | 0.445 |
| TCH, median (IQR), mmol/L | 6.27, (5.765, 7.31) | 5.83, (5, 6.283) | 0.067 |
| HDL, median (IQR), mmol/L | 51.7, (46.25, 58.6) | 1.345, (1.018, 1.648) | 0.031 |
| GLU, median (IQR), mmol/L | 4.33, (4.19, 4.65) | 4.4,(3.98,4.65) | 0.811 |
| TCA, median (IQR) | 0.1291, (0.05812, 0.1532) | 0.3242, (0.1849, 0.3541) | 0.007 |
| TUDCA, median (IQR) | 0.002615, (0.001616, 0.004364) | 0.0021, (0.0010, 0.0041) | 0.276 |
| TCDCA, median (IQR) | 0.09253, (0.07644, 0.1471) | 0.1533, (0.0632, 0.3844) | 0.032 |
| TDCA, median (IQR) | 0.05096, (0.0241, 0.06788) | 0.0505, (0.0172, 0.0719) | 0.707 |
| TLCA, median (IQR) | 0.000505, (0.000153, 0.000829) | 0.00044, (0.00004, 0.00130) | 0.154 |
| GCA, median (IQR) | 0.1847, (0.1026, 0.2427) | 0.2153, (0.0734, 0.4085) | 0.925 |
| GUDCA, median (IQR) | 0.007229, (0.003824, 0.01324) | 0.002563, (0.001693, 0.005983) | 0.956 |
| GCDCA, median (IQR) | 0.1487, (0.113, 0.2245) | 0.1133, (0.0700, 0.1768) | 0.091 |
| GDCA, median (IQR) | 0.08843, (0.0254, 0.1348) | 0.02337, (0.00638, 0.10330) | 0.070 |
| GLCA, median (IQR) | 0.000404, (0.000163, 0.001332) | 0.00006, (0.00005, 0.00018) | 0.131 |
| CA, median (IQR) | 0.04456, (0.003135, 0.1998) | 0.00575, (0.00146, 0.01780) | 0.065 |
| UDCA, median (IQR) | 0.008039, (0.004119, 0.01039) | 0.00103, (0.00070, 0.00402) | 0.055 |
| CDCA, median (IQR) | 0.02225, (0.007754, 0.0908) | 0.00658, (0.00188, 0.01224) | 0.067 |
| DCA, median (IQR) | 0.03327, (0.01601, 0.04815) | 0.01165, (0.002733, 0.02165) | 0.133 |
| LCA, median (IQR) | 0.00025, (0.000118, 0.000912) | 0.00009, (0.00004, 0.00064) | 0.974 |
| Total G, median (IQR) | 0.503, (0.3165, 0.6055) | 0.3817, (0.1883, 0.6611) | 0.263 |
| Total T, median (IQR) | 0.2749, (0.1755, 0.3662) | 0.3677, (0.2345, 0.7563) | 0.012 |
| Total conju, median (IQR) | 0.8527, (0.6435, 0.9537) | 0.9631, (0.9518, 0.9832) | 0.060 |
| Total unconju, median (IQR) | 0.1473, (0.04632, 0.3566) | 0.0369, (0.0168, 0.0482) | 0.060 |
| G/T, median (IQR) | 1.699, (0.8791, 2.32) | 0.6694, (0.2369, 2.2091) | 0.059 |
| Conju/Unconju, median (IQR) | 5.79, (1.805, 20.59) | 26.09, (19.76, 58.64) | 0.030 |
| CA/CDCA, median (IQR) | 1.864, (0.5348, 3.014) | 0.8195, (0.7475, 2.943) | 0.493 |
| DCA/CA, median (IQR) | 0.4301, (0.1904, 9.706) | 1.287, (0.8172, 5.374) | 0.676 |
| LCA/CDCA, median (IQR) | 0.01444, (0.00181, 0.1145) | 0.01337, (0.007052, 0.2773) | 0.322 |
| TBA nmol/L, median (IQR) | 13, (10, 16) | 14, (11, 18) | 0.228 |

Table S4. Preterm birth predict model of EICP.

|  | 95% Confidence interval | Area under the ROC curve (AUC) | *P* value |
| --- | --- | --- | --- |
| ALB | 0.586 to 0.847 | 0.763 | 0.002 |
| TB | 0.556 to 0.812 | 0.692 | 0.049 |
| DB | 0.521 to 0.788 | 0.664 | ns |
| IDB | 0.550 to 0.812 | 0.692 | ns |
| TBA | 0.589 to 0.842 | 0.729 | 0.006 |
| GCA | 0.589 to 0.842 | 0.729 | 0.016 |
| ALB +TBA | 0.683 to 0.907 | 0.813 | <0.001 |
| ALB+TB | 0.654 to 0.889 | 0.788 | <0.001 |
| ALB+GCA | 0.659 to 0.891 | 0.792 | <0.001 |
| ALB+TBA+TB | 0.705 to 0.921 | 0.833 | <0.001 |
| ALB+TBA+GCA | 0.618 to 0.906 | 0.812 | <0.001 |
| ALB+TB+GCA | 0.687 to 0.910 | 0.817 | 0.002 |
| ALB+TB+TBA+GCA | 0.707 to 0.922 | 0.835 | <0.001 |
| TBA+TB | 0.665 to 0.896 | 0.798 | <0.001 |
| TBA+GCA | 0.620 to 0.865 | 0.758 | 0.004 |
| TBA+GCA+TB | 0.676 to 0.903 | 0.808 | <0.001 |
| TB+GCA | 0.652 to 0.887 | 0.787 | <0.001 |

Table S5. Preterm birth predict model of LICP.

|  | 95% Confidence interval | Area under the ROC curve (AUC) | *P* value |
| --- | --- | --- | --- |
| CR | 0.564 to 0.915 | 0.774 | 0.039 |
| HDL | 0.610 to 0.945 | 0.819 | 0.006 |
| Total T | 0.603 to 0.933 | 0.805 | 0.003 |
| TCA | 0.761 to 0.993 | 0.932 | <0.001 |
| TCDCA | 0.444 to 0.828 | 0.654 | ns |
| UREA | 0.551 to 0.908 | 0.762 | 0.027 |
| URIC | 0.573 to 0.926 | 0.787 | 0.023 |
| TCA+HDL | 0.761 to 0.993 | 0.932 | <0.001 |
| TCA+UREA | 0.751 to 0.993 | 0.929 | <0.001 |
| TCA+URIC | 0.755 to 0.995 | 0.935 | <0.001 |
| TCA+CR | 0.741 to 0.990 | 0.921 | <0.001 |
| ALB+TB+TBA+GCA | 0.707 to 0.922 | 0.835 | <0.001 |
